# Lymphatics in Tumor Progression and Immunomodulation

**DOI:** 10.3390/ijms23042127

**Published:** 2022-02-15

**Authors:** Claire Y. Li, Stav Brown, Babak J. Mehrara, Raghu P. Kataru

**Affiliations:** The Department of Surgery, Division of Plastic and Reconstructive Surgery, Memorial Sloan Kettering Cancer Center, New York, NY 10065, USA; yul9025@nyp.org (C.Y.L.); brownrs@mskcc.org (S.B.); mehrarab@mskcc.org (B.J.M.)

**Keywords:** lymphatics, metastasis, tumorigenesis, tumor microenvironment, immunomodulation

## Abstract

The lymphatic system consists of a unidirectional hierarchy of vessels responsible for fluid homeostasis, lipid absorption, and the transport of immune cells and antigens to secondary lymphoid organs. In cancer, lymphatics play complex and heterogenous roles that can promote or inhibit tumor growth. While lymphatic proliferation and remodeling promote tumor dissemination, functional lymphatics are necessary for generating an effective immune response. Recent reports have noted lymphatic-dependent effects on the efficacy of immunotherapy. These findings suggest that the impact of lymphatic vessels on tumor progression is organ- and context-specific and that a greater understanding of the interaction of tumor cells, lymphatics, and the tumor microenvironment can unveil novel therapies.

## 1. Introduction

Tumor metastasis is the leading cause of cancer-related mortality and requires the dissemination of tumor cells from the primary tumor into the systemic circulation [1]. Tumor cells can reach the systemic circulation through either hematogenous or lymphatic routes [1]. In the case of the latter, tumor cells traverse the lymphatic system consisting of a hierarchical network that functions in the unidirectional drainage of interstitial fluid, lipids, and cells from the peripheral tissue to upstream lymph nodes [2]. While lymphatic capillaries are composed of a single layer of lymphatic endothelial cells (LECs) with discontinuous, button-like junctions to facilitate fluid and cellular entry into the lumen, collecting lymphatics have continuous zipper-like junctions with a basement membrane and a contractile smooth muscle layer to facilitate the pumping of lymph upstream into lymph nodes [3,4,5]. The hierarchy of lymph nodes eventually drain into the central venous system via the thoracic duct [6].

Despite a myriad of studies detailing the biological importance of angiogenesis in the tumor setting, the study of tumor lymphangiogenesis has lagged behind until about 2 decades ago, when the identification of lymphatic specific markers such as transcription factor Prox-1 and surface proteins LYVE-1, FLT4 (VEGFR3), and podoplanin accelerated our understanding of lymphatic biology both in the physiologic and pathologic settings [2,6]. Specifically, in the context of cancer, recent reports suggest that many of the physiologic functions of lymphatic vessels are co-opted by tumor cells for their own growth and metastasis [1,2,7]. In the tumor microenvironment (TME), lymphatics undergo numerous changes including proliferation, elaboration of chemotactic molecules, and induction of immunomodulatory functions that influence tumorigenesis and metastasis [2]. In addition, the anatomy of lymphatic capillaries also promotes initial tumor dissemination given its permeable nature [8]. Evidence from several studies has established a link between lymphangiogenesis and metastasis, supporting the hypothesis that increased access to lymphatic capillaries raises the likelihood of tumor cell intravasation and metastasis [9]. Interestingly, lymphangiogenesis in premetastatic lymph nodes or distant sites promotes metastasis in these areas by establishing a “lymphovascular niche”, highlighting the fact that the tumorigenic effects of lymphatics extend beyond simply increased surface area for tumor cell invasion [10,11]. Tumor cells also hijack chemotactic gradients produced by LECs that aid in physiologic leukocyte homing to drive tumor cell migration [8]. In addition to the well-established roles of lymphatics in transporting fluid, macromolecules, and immune cells, recent studies have also shown that LECs can act as antigen-presenting cells at steady state and that this function can be co-opted by tumor cells to promote immune-evasion [12,13,14,15].

**Figure 1:**
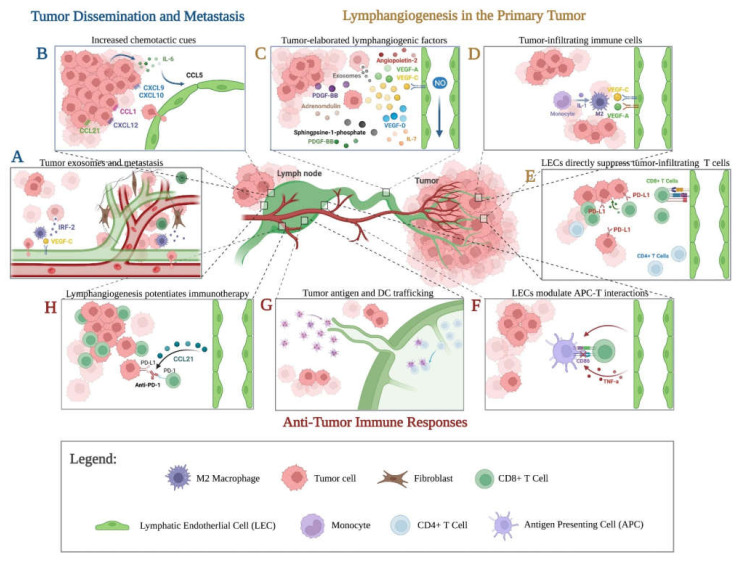
The role of lymphatics in tumor progression and immunomodulation. (**A**) Tumor invasion of nodal lymphatics results in lymphatic spread while invasion of lymph node blood vessels leads to systemic dissemination. Tumor exosome IRF-2 increases lymph node lymphangiogenesis by inducing the secretion of VEGF-C by nodal macrophages. (**B**) Various chemotactic cues in tumor-draining lymph nodes promote tumor entry and colonization of the lymph node. (**C**) Tumor-elaborated lymphangiogenic factors mediate intratumoral and peritumoral lymphatic expansion. VEGF-C acts on its receptors VEGFR-2/3 to induce tumoral lymphangiogenesis through the activation of endothelial-derived nitric oxide synthase (eNOS) by LECs, leading to increased lymphatic flow around the tumor and facilitating lymphatic metastasis. Additional tumor-elaborated lymphangiogenic factors include VEGF-D, VEGF-A, PDGF-BB, angiopoietin-2, sphingosine-1-phosphate, adrenomedullin, IL-7, and exosome-derived molecules. (**D**) Tumor-infiltrating immune cells secrete molecules involved in lymphatic restructuring; Tumor-derived IL-1 promotes lymphangiogenesis and lymph node metastasis through polarization of M2 macrophages that secrete VEGF-A and VEGF-C. (**E**) Lymphatics act as immunosuppressive cells in the tumor microenvironment. IFNγ elaborated by tumor-specific CD8+ T cells induces expression of lymphatic PD-L1, which limits local CD8+ T-cell tumor infiltration. (**F**) LECs modulate Antigen Presenting Cells (APC)-T-cell interactions. TNFα-stimulated LECs reduce the ability of dendritic cells to induce T-cell proliferation through reduction of CD86 expression by dendritic cells in a MAC-1/ICAM-1-dependent manner. (**G**) Impaired lymphatic transport from dysfunctional peritumoral lymphatics hampers the generation of an effective adaptive immune response. Generation of the anti-tumor response occurs in the sentinel lymph node, whereby dendritic cells present tumor antigens to prime naïve T lymphocytes. (**H**) VEGF-C-induced CCL21 upregulation in tumor lymphatics potentiates adoptive T-cell therapy. VEGF-C mediated lymphangiogenesis results in improved response to anti-PD-1/CTLA-4 combination therapy, which is dependent on the CCL21/CCR7 pathway.

In contrast to previous views of lymphatics as passive conduits for tumor and immune cells, we now understand that lymphatics also play active and complex roles in tumor progression and anti-tumor immunity. In this review, we discuss our current understanding of the development of tumor lymphatics as well as its role in primary tumor progression, metastasis, and modulation of the host immune response (Figure 1).

## 2. Cellular Origins of Tumor-Associated Lymphatics

In the setting of inflammation, lymphatics proliferate [16]. Although this is also observed in the TME, the cellular origins of tumor-associated LECs are still debated [17]. While He and colleagues showed that tumor lymphatics are derived exclusively from pre-existing lymphatics, other studies suggest nonendothelial cellular origins, mainly that of bone marrow (BM) origin [18]. For example, using irradiated mice reconstituted with enhanced green fluorescent protein (EGFP)-positive donor BM cells, Religa et al. showed that there were EGPF+ LYVE1+ cells in the peritumoral lymphatics of heterotopically implanted fibrosarcoma tumors [19]. These progenitor cells only comprise a small percentage of the neo-lymphatic vasculature in the TME, found to be about 3–4% in one study [20]. Additionally, mice bearing heterotopic tumors injected with ex vivo isolated BM-derived podoplanin+ showed incorporation of injected cells into the peritumoral lymphatics as well as increased numbers of these cells in the BM and peripheral blood, suggesting activation and mobilization of these putative progenitors during tumorigenesis [21].

More recent studies have focused on the contribution of BM-derived cells (BMDCs) of the myeloid lineage on tumor lymphangiogenesis. Using BM transplantation and genetic lineage-tracing experiments, Zumsteg and colleagues showed that BMDCs can integrate into the tumor lymphatics of the Rip1Tag2 mouse model of insulinoma and in the TRAMP-C1 prostate cancer transplantation models, and that these progenitor cells originate from the myeloid lineage [22]. Analysis of human breast cancer specimen showed increased expression of myeloid and stem cell markers in peritumoral lymphatics compared to normal breast tissue lymphatics, thereby supporting animal studies demonstrating the contribution of myeloid stem-cell to tumor lymphatics [23]. Subsequently, inhibition of myeloid differentiation and recruitment was associated with reduced tumor lymphatic density [23]. Myeloid-lymphatic transition appears to be dependent on Toll-Like Receptor 4 (TLR4); stimulation of human and mouse myeloid cells by TLR4 ligands induced lymphatic-specific genes such as LYVE-1, podoplanin, and VEGFR3 [24]. Additionally, these TLR4-reprogramed myeloid LEC progenitors generated functional in vivo lymphatics in orthotopic breast cancer models [24]. However, contrasting results regarding the contribution of myeloid progenitors to tumor lymphatics were reported by Gordon et al., in which LysM:Cre mice and lineage tracing experiments demonstrated that, while F4/80-positive cells appeared to be integrated within tumor lymphatics, these cells did not express PROX1 [25]. The authors argue that this shows that LEC progenitors arise independently of the myeloid cell lineage in the TME [25]. Overall, the origins of LECs in the TME remain an area of debate. Although several lines of evidence support a minor contribution of BMDCs to tumor neo-lymphangiogenesis, its contribution to tumor progression and metastatic spread remain unclear.

## 3. Lymphangiogenesis in the Primary Tumor

Tumor lymphangiogenesis is a feature of many solid tumors; indeed, tumor lymphatic density is correlated with increased incidence of lymph node metastasis in several human cancers, including head and neck cancer [26], prostate cancer [27], hilar cholangiocarcinoma [28], cervical carcinoma [29], and melanoma [30]. However, in colorectal cancer, low lymphatic density—as well as markers of decreased immune cytotoxicity—are correlated with increased metastatic potential, suggesting tumoral lymphangiogenesis may have anti-tumor roles in the activation of the host immune response [31]. These findings underscore the organ- and context-specific roles of local lymphatics in the regulation of tumor progression.

Early investigations on the biological significance of tumoral lymphatics focused on soluble lymphangiogenic factors found in abundance in the TME (Table 1). Intratumoral and peritumoral lymphatic expansion is mediated primarily by vascular endothelial growth factor (VEGF)-C, and the expression of this growth factor is also associated with increased lymph node metastasis [32,33,34]. VEGF-C acts on its receptors VEGFR-2/3; inhibition of VEGFR-3 signaling decreases tumor lymphangiogenesis and sentinel node metastasis in VEGF-C expressing tumors [35,36,37]. One mechanism by which VEGFR-2/3 activation induces tumoral lymphangiogenesis is through the activation of endothelial-derived nitric oxide synthase (eNOS) by LECs [38]. Additionally, VEGF-C elaborated by cancer cells is associated with increased lymphatic flow around the tumor, which is hypothesized to facilitate lymphatic metastasis [39]. Aside from VEGF-C, VEGF-D and VEGF-A have also been implicated in increased intratumoral lymphangiogenesis and lymph node metastasis [40,41]. Lymphangiogenic factors outside of the VEGF family involved in tumor lymphangiogenesis include PDGF-BB [42], angiopoietin-2 [43], sphingosine-1-phosphate [44], adrenomedullin [45], and IL-7 [46]. More recently, tumor exosome-derived molecules, including noncoding RNA and miRNA, have been reported to increase tumor lymphangiogenesis through modulation of lymphangiogenic gene expression in LECs [47,48,49].

In addition to tumor-elaborated lymphangiogenic factors, tumor-infiltrating immune cells also secrete molecules involved in lymphatic restructuring. Tumor-derived IL-1 promotes lymphangiogenesis and lymph node metastasis through polarization of M2 macrophages that secrete VEGF-A and VEGF-C [50]. M2 macrophages injected into heterotopically implanted 4T1 murine breast cancer tumors promoted primary tumor growth and lung metastases, and this phenotype was associated with increased expression of VEGF-A, VEGF-C, LYVE-1, HIF-1alpha, and CD31 in vivo [53]. Similarly, co-culture of 4T1 tumor cells and M2 macrophages in vitro have induced expression of various inflammatory, lymphangiogenic, and angiogenic cytokines [53].

The effects of local lymphangiogenesis on primary tumor growth are not well characterized and conflicting findings have been reported, emphasizing the heterogeneity of tumor lymphatic function and potential differences in the definition of intratumoral versus peritumoral lymphatics used in various studies. In a heterotopic and spontaneous mammary tumor model in mice, suppression of peritumoral LEC proliferation decreased rates of lymph node and lung metastasis but not primary tumor growth [54]. In contrast, primary tumor growth was increased in transgenic mice (kCYC) with dermal lymphatic dysfunction; this phenotype was due, at least in part, to decreased tumor immunity secondary to impaired antigen presentation [55]. Although additional studies are clearly needed, based on the available data, it appears that the biological significance of the lymphatic system on primary tumor growth is relatively minimal. In contrast, a wealth of studies suggests that local tumor lymphangiogenesis plays a key role in lymphatic dissemination, which will be discussed next.

## 4. Lymphatics in Tumor Dissemination

While the prognostic significance of lymph node metastasis has been established in many solid tumors, the ability of these sites of metastasis to contribute to further dissemination has been historically debated given the limited survival benefit of lymphadenectomy in many cancers [1,7]. However, recent lineage tracing studies in human colorectal and prostate cancer demonstrate that tumor cells from metastatic lymph nodes can contribute to distant metastasis, confirming that the lymphatic system represents a major route for tumor dissemination [56,57]. Because of the link between peritumoral lymphangiogenesis and lymphatic metastasis, local lymphangiogenesis is hypothesized to promote lymphatic metastasis by increasing tumor cell access to lymphatic vessels, although the exact molecular mechanism is not well defined [1]. Analysis of human melanoma specimens using electron microscopy shows single tumor cells invading the lymphatic capillaries by penetrating the subendothelial space [58]. Debate also exists regarding the biological importance of intratumoral versus peritumoral lymphatics in the development of metastasis. Padera and colleagues first noted that peritumoral lymphatics were dilated with open lumens, compared to intratumoral lymphatics that had collapsed lumens [59]. They, and others, have shown that intratumoral lymphatics are neither functional nor necessary for lymphatic metastasis [60]. 

Once inside the lymphatic system, tumor cells encounter an environment that further promotes tumor survival and invasion of the upstream lymph node. For example, one recent study showed higher levels of glutathione and oleic acid and decreased levels of free iron in lymph fluid which protected melanoma cells from ferroptosis, thereby increasing their capacity to form metastasis compared to cells in blood vessels [61]. Additionally, Lee and colleagues found that bioactive bile acids accumulate in metastatic lymph nodes, which subsequently activate the transcriptional coactivator yes-associated protein (YAP); YAP induces upregulation of fatty-acid oxidation signaling in tumor cells to shift their metabolism toward fatty acid oxidation, which promotes survival in lymph node microenvironments [52]. Additionally, dilation of the collecting lymphatic vessel draining the primary tumor occurs prior to lymph node metastasis and is associated with increased lymphatic flow [10,51]. These structural changes are believed to increase lymph drainage from the primary tumor to the upstream lymph node. Interestingly, after lymph node metastasis, lymph flow decreases and can even reroute to other non-sentinel draining lymph nodes through collateral lymphatics [62,63]. Phenotypic changes also occur in lymphatics in the form of increased chemotactic cues that further promote lymphatic metastasis. For example, tumor CXCR3 and tumor-draining lymph node CXCL9 and CXCL10 promote the metastasis of murine melanoma cell line B16F10 [64]. Lee and colleagues demonstrated that tumor elaboration of IL-6 induced Stat3 phosphorylation in lymph node LECs, resulting in increased expression of CCL5 that facilitated metastasis [65]. The CCL21-CCR7 axis has also been shown to be an important lymphatic regulator of metastasis; tumor expression of CCR7 increases lymph node metastasis in mouse models, while neutralization of the CCR7 ligand CCL21 in lymphatics decreases lymph node metastasis [66,67]. Using human melanoma and breast cancer cell lines implanted in mice, Das and colleagues found that CCL1 expression on lymph node sinuses controls initial tumor entry into the lymph node; neutralization of CCR8, the receptor for CCL1, led to the sequestration of tumor cells at the junction of the collecting lymphatic vessel and the lymph node subcapsular sinus [68]. In addition to the CCL1-CCR8 axis, the CXCL12/SDF-1-CXCR4 axis has also been implicated as a critical checkpoint for the entry of metastasis into the lymph node [69]. 

In addition to the increased expression of chemokines and cytokines in the premetastatic lymph node, lymphangiogenesis in the node and distant sites also appears to contribute to increased metastasis [10,11,41,70]. Lymphangiogenesis in these sites occurs before metastasis, and inhibition of lymph node lymphangiogenesis decreases nodal tumor growth in a rat breast cancer model [10,11,71]. However, the mechanism of how lymphangiogenesis contributes to metastatic progression has yet to be elucidated. In 1889, Stephen Paget first proposed the concept of the “seed and soil” theory of tumor metastasis in which tumors arriving at metastatic sites only occurred when the appropriate premetastatic conditions (“the soil”) were achieved [72]. Therefore, one existing hypothesis is that lymphangiogenesis in the draining lymph nodes or distant sites is the result of the tumor conditioning the “soil.” Recent studies have demonstrated that tumor-secreted molecules induce lymphangiogenesis in the premetastatic node (Table 1). For example, tumor exosomes condition sentinel lymph nodes by increasing extracellular matrix deposition and vascular proliferation that promote tumor recruitment and progression [73]. Additionally, tumor exosome IRF-2 has been found to increase lymph node lymphangiogenesis by inducing the secretion of VEGF-C by nodal macrophages [74]. Using a VEGFR3 lymphatic reporter, Olmeda and colleagues tracked distant site lymphangiogenesis and showed that this effect was uncoupled from primary tumor lymphangiogenesis [75]. This strategy identified midkine—a tumor-elaborated soluble factor—as a mediator of distant lymphangiogenesis [75]. Analysis of human melanoma and breast cancer specimens with metastatic sentinel nodes show that lymph node lymphangiogenesis after tumor nodal colonization is associated with additional nodal metastasis [76,77]. Interestingly, in a study of human melanoma sentinel lymph node metastasis, sentinel node lymphangiogenesis was associated with additional nodal metastasis, while sentinel node angiogenesis was associated with distant organ metastasis [77]. These findings suggest two mechanisms for further tumor dissemination from the sentinel lymph node: 1. invasion of lymph node lymphatics leads to further lymphatic spread and 2. invasion of nodal blood vessels results in systemic progression [77]. In support of this hypothesis, two independent laboratories recently showed that tumor cells in the lymph node can escape through high endothelial venules to reach distant organs in mice [78,79]. Notably, these two studies provided the first preclinical evidence that tumors cells can be disseminated directly into the systemic circulation from lymph nodes, thereby bypassing the thoracic duct. Taken together, recent evidence shows that tumor-secreted factors induce lymphangiogenesis in upstream lymph nodes, which is associated with increased metastasis; the mechanisms of how lymph node lymphangiogenesis may lead to increased metastasis may be that of increased tumor extravasation from lymph nodes into the systemic circulation. 

## 5. Lymphatics and the Anti-Tumor Immune Response

Lymphatics serve both indirect and direct roles in the modulation of the host adaptive immune response through transport of immune cells and tumor antigens to the lymph node and direct immunomodulation, respectively. Priming of the anti-tumor response occurs in the sentinel lymph node, a process that relies on the presentation of tumor antigens by type 1 and 2 conventional dendritic cells to prime lymphocytes [80]. Aberrations in effective lymphatic transport in the form of dysfunctional peritumoral lymphatics hamper the generation of an effective adaptive immune response [55,81]. Tumors implanted in K14-VEGFR3-Ig transgenic mice lacking dermal lymphatics due to the expression of a soluble VEGFR3 have impaired tumor antigen and dendritic cell trafficking to lymph nodes in addition to impaired anti-tumor immunity in response to dermal vaccine [81]. Similarly, local lymphatic ablation in a LEC-specific transgenic mouse model resulted in decreased numbers of tumor-infiltrating lymphocytes and overall immunosuppressive milieu [82]. Analysis of colorectal patients shows that decreased lymphatic density and immune cytotoxicity in the TME were predictive of metastasis rather than tumor-intrinsic factors such as chromosomal instability or cancer-associated mutations [31]. All in all, these studies suggest that functional lymphatics in the TME hamper tumor progression to metastasis through the generation of an effective immune response. 

Aside from the trafficking of tumor-antigen carrying dendritic cells, emerging evidence highlights direct immunomodulatory roles for LECs in tumorigenesis. The tolerogenic potential of LECs has been most extensively studied at steady state; LECs promote peripheral tolerance through the presentation of peripheral tissue antigens on major histocompatibility class (MHC) molecules to suppress self-reactive lymphocytes [12,13]. Disruptions in this pathway result in signs of autoimmunity in mice [12]. There is recent evidence to suggest that, in the tumor setting, LECs can similarly act as immunosuppressive cell types. For example, lymph node LECs have been shown to suppress antigen-specific CD8+ T cells through cross presentation of exogenous antigens on MHC-I in a murine B16F melanoma model [14]. Intratumoral injection of ex-vivo isolated lymph node podoplanin+ cells, which consists of follicular reticular cells and LECs, promotes tumor growth through the inhibition of tumor-infiltrating CD4+ T lymphocytes [83]. In the TME, IFNγ elaborated by tumor-specific CD8+ T cells induces expression of lymphatic PD-L1, which limits local CD8+ T-cell tumor infiltration in various murine melanoma models and MC38 colon adenocarcinoma [15]. Additionally, lymphatic PD-L1 deficiency was found to result in the expansion of tumor-specific CD8+ T cells in tumor-draining lymph nodes in mouse B16 and MC38 tumor models [84]. 

The direct antigen-presenting capability of LECs has been demonstrated using in vitro experiments in which LECs pulsed with foreign ova peptide enhanced killing of B16F10-ova tumor cells by ova-specific CD8+ T cells [85]. More recently, Gkountidi and colleagues found that LECs in the TME promote regulatory T-cell-suppressive function in an MHC-II-dependent manner [86]. In this study, mice lacking lymphatic specific MHC-II led to locally reduced Treg cell tumor suppression, and as a consequence increased TME infiltration of tumor-killing T effector cells that reduced primary tumor growth [86]. In addition to direct interaction with T cells, LECs also modulate APC-T-cell interactions [87,88]. TNFα-stimulated LECs reduced the ability of dendritic cells to induce T-cell proliferation through reduction of CD86 expression by dendritic cells in a MAC-1/ICAM-1–dependent manner [87]. Additionally, LEC-secreted inhibitory factors, such as indoleamine 2,3 dioxygenase, has been shown to impair dendritic cell-induced T-cell proliferation [88]. All in all, recent evidence suggests that tumor-associated lymphatics have both pro- and anti-tumor immunomodulatory roles through the presentation of tumor antigens to suppress tumor-specific lymphocytes and the transport of tumor antigens to prime lymphocytes in the lymph node, respectively. 

## 6. Targeting Lymphatics for Cancer Therapeutics

Studies targeting lymphatics in the treatment of cancer have focused on two major strategies: inhibition of lymphangiogenic signals to impede lymphangiogenesis or manipulation of the lymphatic system to promote priming of the anti-tumoral immune response. Many of the preclinical animal studies looking into the therapeutic benefits of blocking lymphangiogenic signals focused on inhibition of the VEGF-C/VEGFR3 axis at various levels. Two studies using soluble tumor-excreted sVEGFR3-Ig as a decoy receptor for ligands VEGF-C and VEGF-D showed reduced tumor lymphatic proliferation and lymph node metastasis in mice and rats, respectively [89,90]. Similarly, intravascular administration of adenovirally delivered sVEGFR3-Ig in mouse xenotransplants also inhibited the formation of lymph node macrometastasis in a dose-dependent manner [36]. Studies that employed neutralizing antibodies against VEGFR3 showed reduced tumor-associated lymphatic formation, tumor growth, and lymph node metastasis in mice [91,92,93]; however, monotherapy using a humanized monoclonal anti-VEGFR3 (LY3022856) in a phase I clinical trial of 44 patients showed minimal anti-tumor activity as measured by radiographic response [94]. Lastly, strategies to neutralize VEGFR3 downstream effectors in the form of tyrosine kinase inhibitors have been used extensively for clinical and experimental purposes, although none are specifically designed to target lymphatic pathways [95]. However, SAR131675, a novel VEGFR3-specific tyrosine kinase inhibitor, has been shown to reduce primary tumor growth as well as lymph node and lung metastasis through the inhibition of lymphangiogenesis and tumor-associated macrophages invasion [96]. Currently, no therapeutic strategies targeting VEGF-C/VEGFR3 have been shown to be beneficial for clinical use. 

Aside from targeting lymphangiogenesis, recent findings highlighting the immunomodulatory potential of the lymphatic system have turned attention instead towards harnessing these mechanisms to improve anti-tumor immunotherapy. VEGF-C–induced lymphangiogenesis potentiated adoptive T-cell therapy in a heterotopic B16 melanoma model and anti-PD-1 therapy in a transgenic Braf^V600E^/Pten^−/−^ melanoma mouse model in one study [97]. VEGF-C mediated potentiation of adoptive T-cell therapy was dependent on CCR7-mediated attraction of naïve T cells to the local TME; this was shown to occur through VEGF-C-induced CCL21 upregulation in tumor lymphatics because CCR7 blockade reversed the potentiating effects of VEGF-C [97]. Using heterotopic brain tumor models, Hu et al. similarly found that VEGF-C mediated lymphangiogenesis resulted in improved response to anti-PD-1/CTLA-4 combination therapy and that this effect was dependent on the CCL21/CCR7 pathway [98]. VEGF-C–mediated lymphangiogenesis has also been shown to augment immunotherapy in a glioblastoma mouse model by increasing CD8+ T-cell priming in tumor-draining lymph nodes and promoting egress of tumor-specific CD8+ T cells to the primary tumor [99]. Using a lymphangiogenic vaccine, Sasso and colleagues elegantly showed that local lymphangiogenesis promotes more robust T-cell activation both locally in the TME and in the draining lymph node to provide improved tumor control [100]. 

Based on these animal studies, there is preliminary evidence that VEGF-C-mediated lymphangiogenesis may potentiate immunotherapy in the clinical setting. For example, tumor peptide-specific CD8+ T cell number and functionality are positively correlated with serum VEGF-C concentration in melanoma patients treated with tumor peptide vaccination [97]. Notably, patients treated with combined anti-PD-1 and anti-CTLA-4 checkpoint blockade with high levels of serum VEGF-C have shown significantly longer progression-free survival than patients with lower levels of VEGF-C [97]. Taken together, these recent studies show that manipulation of the lymphatic system to become more functional in the priming of the host immune response may have translational utility. 

## 7. Conclusions and Future Directions

Although recent discoveries in lymphatic biology have shed new insights into the heterogenous effects lymphatics have on tumor progression, metastasis, and immunomodulation, many questions remain unanswered. For one, while lymphangiogenesis in the primary tumor is associated with increased lymphatic metastasis in certain tumors, the mechanism driving initial tumor cell invasion of precapillary lymphatics remains unclear. Whether it is increased access to the lymphatic endothelium during lymphangiogenesis or increased lymphatic leakiness in the TME that promotes initial tumor invasion is still unknown. The relative contribution of blood versus lymphatic vessels for tumor escape from the sentinel lymph node also requires additional clarification. Furthermore, while the association of sentinel lymph node lymphangiogenesis on lymphatic metastasis has been demonstrated in several clinical studies, the molecular underpinnings of this observation have yet to be reported. Using gene-expression profiling techniques, one recent study found that LECs in tumor draining lymph nodes upregulate integrin αIIb expression which mediates LEC adhesion to fibrinogen [101]. It is possible that this and other LEC surface proteins promote tumor cell chemotaxis into draining lymph node. Moreover, the consequences of lymph node removal, either by surgical resection or lymph node metastasis, on immunotherapy, prompts clarification. One recent report showed that immune checkpoint blockade administered directly into primary or secondary tumor draining lymph nodes improved tumor-specific T-cell priming compared to therapy administered systemically; this result not only suggests novel approaches to potentiate immunotherapy, but also underscores the role of tumor draining lymph nodes as survival niches for lymphocyte priming [102]. Therefore, as more and more solid tumors are being treated with immunotherapy, the consequences of lymph node removal on the efficacy of immunotherapy warrants additional investigation.

Arguably the most promising in terms of the translational utility of lymphatic biology for cancer therapeutics is the discovery of the immunomodulatory potential of the lymphatic system. The ability of LECs to present peripheral tissue antigens to mediate tolerance at steady state extends to the cancer setting in which LECs present tumor antigen to induce immunosuppressive phenotypes in lymphocytes [14]. This immunosuppressive role LECs play in the lymph node, and possibly locally in the primary tumor, contrasts with the potentiating effects lymphangiogenesis has on immunotherapy, as reported by Fankhauser et al. and Song et al.; these findings again highlight the complex and heterogenous roles lymphatics play in tumorigenesis, which will inevitably complicate future clinical translation [97,99]. Continued research into the mechanisms of lymphatic metastasis and the immunomodulatory capabilities of the lymphatic system will further our understanding of how lymphatics can be manipulated for therapeutics.

## Figures and Tables

**Table 1:** Summary of tumor-secreted molecules affecting lymphatic phenotype.

Molecule	Target cell	Target Pathway	Function Tumor	Origin	Refs
Cytokines					
IL-1	MФ	IKKβ/NF-кB	Recuitment and activation of lymphangiogenic M2 MФ	Lung	[36]
IL-6	LECs	pStat3-pcJun-pATF-2 ternary complex	Increased lymphatic chemctactic cues, increased lung vascular permeability, increased LN angiogenesis	Breast	[50]
IL-7	Tumor ;	cFos and c-Jun heterodimer	Increased tumor lymphangiogenic	Lung	[32]
Grouth factors					
VEGF-A	LECs	-	Increased tumor lymphangiogenic, increased LN angiogenesis	Cutaneous SCC	[27]
VEGF-C	LECs	eNOS	Increased tumor lymphangiogenic, increased LN angiogenesis	Breast, pancreas, prostate, lung, melanoma, fibrosarcoma	[18,19,20,21,22,23,24]
VEGF-D	LECs	Prostaglandin production	Increased tumor lymphangiogenesis, dilation of collecting efferent lymphatic vessel	Breast	[26,46]
PDGF-BB	LECs	MAP Kinases Erk1/2 and Akt	Increased tumor lymphangiogenic	fibrosarcoma	[28]
Exosome-derived					
Long noncoding RNALINC00858	LECs	Prax-1 transcription	Increased tumor lymphangiogenic	Bladder	[33]
Long noncoding RNASNHG16	LECs	SOX18 transcription	Increased tumor lymphangiogenic	Bladder	[34]
miRNA-221-3p	LECs	VASHI/ERK/AKT	Increased tumor lymphangiogenic	Cervical SCC	[35]
IRF-2 M	MФ	-	Increased LN angiogenesis through MФ secreted VEGF-C	Colorectal carcinoma	[51]
Other					
Adrenomedullin	LECs	-	Increased tumor lymphangiogenic	Lung	[31]
	LECs	mTOR	Increased tumor lymphangiogenic, increased LN angiogenesis, increased distant organ lymphangiogenesis	Melanoma	[52]

IL, interleukin; MΦ, macrophage; NF-κB, NF-kappaB; LECs, lymphatic endothelial cells; pStat-pc-Jun-pATF, phosphorylated Stat-pc-Jun-phosphorylated activated transcription factor-2; LN, lymph node; VEGF, vascular endothelial growth factor; eNOS, endothelial nitric oxide synthase; PGDF-BB, platelet-derived growth factor-BB; MAP, mitogen-activated protein; Erk, extracellular signal-regulated kinases; Akt, protein kinase B; miRNA, microRNA; VASH1, vasohibin-1; IRF-2, interferon regulatory factor-2; mTOR, mechanistic target of rapamycin.

## Data Availability

Not applicable.
